# Overweight, Obesity, and Musculoskeletal Symptom Burden in Chile: A Population-Based Cross-Sectional Study

**DOI:** 10.3390/healthcare14203369

**Published:** 2026-10-09

**Authors:** Pablo A. Lizana, Camila Lamas-Cirano, Ana Muñoz-Cáceres, Rodrigo Lizama-Pérez, Rodrigo Muñoz-Cofré

**Affiliations:** 1 Laboratory of Epidemiology and Morphological Sciences, Instituto de Biología, Pontificia Universidad Católica de Valparaíso, Valparaíso 2373223, Chile; camila.lamas.c@mail.pucv.cl (C.L.-C.); ana.munoz.c@mail.pucv.cl (A.M.-C.); 2 Center for Interdisciplinary Research in Biomedicine, Biotechnology and Well-Being (CID3B), Pontificia Universidad Católica de Valparaíso, Valparaíso 2373223, Chile; 3 Departamento de Anatomía Normal y Medicina Legal, Facultad de Medicina, Universidad de Concepción, Concepción 4070386, Chile; rlizama@udec.cl; 4 Centro de Excelencia en Estudios Morfológicos y Quirúrgicos, Universidad de La Frontera, Temuco 4811230, Chile; rodrigo.munoz@ufrontera.cl

**Keywords:** musculoskeletal symptoms, body mass index, overweight, obesity, population health, health surveys, Chile

## Abstract

**Background/Objectives:** Musculoskeletal symptoms (MSS) are among the leading causes of pain and functional limitation worldwide, yet population-level evidence linking excess body weight to MSS in Latin America remains scarce. This study examined the association between body mass index (BMI) and the prevalence of non-traumatic MSS in the Chilean population. **Methods:** A cross-sectional secondary analysis was conducted using data from the 2009–2010 Chilean National Health Survey (n = 4689). MSS were assessed with the validated COPCORD CCQ-ILAR questionnaire, covering 12 anatomical regions over the previous seven days. BMI was categorized as <25, 25 to <30, and ≥30 kg/m^2^. Survey-weighted negative binomial and logistic regression models were fitted, adjusting for age, sex, educational level, and area of residence. **Results:** Over half the sample (55.26%) reported at least one MSS. Each one-unit increase in BMI was associated with a 2.8% higher expected symptom count (CR = 1.028; 95% CI: 1.019–1.037). Compared with participants with BMI < 25 kg/m^2^, those with overweight and obesity had 55.7% and 98.4% higher odds of reporting ≥1 MSS, respectively. The knee, shoulder, lower back, and ankle showed the steepest prevalence gradients across BMI strata. After correction for multiple comparisons, overweight remained associated with lower-back, finger, and knee symptoms, whereas obesity remained associated with shoulder, wrist, finger, knee, and ankle symptoms. Age and female sex were also independently associated with greater symptom burden. **Conclusions:** Higher BMI was positively associated with a greater overall MSS burden. Region-specific analyses identified robust associations in selected weight-bearing and upper-limb regions after correction for multiple comparisons. Because of the cross-sectional design, these findings do not establish that reducing BMI would reduce MSS. In addition, as the data were collected in 2009–2010, the prevalence estimates should not be interpreted as representing the current MSS burden in Chile.

## 1. Introduction

Musculoskeletal symptoms (MSS) encompass a range of conditions affecting muscles, bones, joints, tendons, ligaments, and other connective tissues [1]. These conditions can range from acute issues, such as sprains and fractures, to chronic disorders that progressively diminish mobility, functional capacity, and overall quality of life. This pain not only limits individuals’ movement but also significantly impairs their quality of life [2,3].

The prevalence of MSS has been studied across different groups, particularly the working population [4]. In this regard, different prevalences of MSS by body segment have been reported. In the Netherlands, for example, it has been reported that 50% of the working population with a normal body mass index (BMI) had MSS [5]. In computer workers, researchers documented a higher frequency of MSS in the lower back, neck, and shoulders [6]. Similarly, in the teaching population, a high prevalence of MSS has been documented in the same areas [7,8,9,10], as well as in the pelvis and lower limb [11,12]. Therefore, the background indicates the relevance of studying MSS in the population, not only to identify its prevalence but also to examine its association with other factors that may indicate a risk for MSS development.

Another health condition that has been studied in relation to MSS is overweight and obesity. Previous studies have reported a greater prevalence and burden of MSS and pain among individuals with elevated BMI, with particularly consistent associations in weight-bearing regions such as the knee and lower back [5,13,14,15,16,17,18]. These findings support the hypothesis that excess body weight may be associated with MSS through both mechanical and systemic pathways, although the magnitude and anatomical distribution of these associations vary across populations. Overweight and obesity are characterized by excess body fat that may adversely affect health [19,20]. The parameter most studied for determining nutritional status is BMI. However, currently, it has been suggested to consider other parameters that reflect body composition in a more detailed way, such as the percentage of body fat or muscle mass, because this can differ according to biological sex, as well as age, so authors point out that BMI use may lead to misclassification of the nutritional status for an individual [21,22].

In Chile, MSS have been studied as a risk factor for individuals’ quality of life. Through a secondary analysis of the results of the 2009–2010 National Health Survey (NHS) of Chile, it has been reported that chronic MSS (present for 90 days or more), whose prevalence in the surveyed population was 42.6% (95% CI: 40.4–44.9), is a risk factor for health-related quality of life in its physical and mental components, together with a low educational level and advanced age, which was mainly observed in women [3]. In addition, another study focused on a group of Chilean workers, such as teachers, found a high prevalence of MSS, with a higher prevalence in the lower back. However, considering age, those younger than 44 years had a higher prevalence of MSS in the upper limb, while those older than 44 years had a higher prevalence in the lower limb [7]. During the COVID-19 pandemic, 98% of teachers in Chile reported experiencing some level of MSS [23]. This background highlights the importance of reporting results at the national level and the role of risk factors, such as obesity, in preventing MSS in the long term.

The Chilean population has consistently shown a high prevalence of overweight and obesity, with particularly high levels reported among women and middle-aged adults [24]. At the same time, previous Chilean studies have documented a high burden of MSS, including chronic musculoskeletal symptoms associated with poorer health-related quality of life in the general population [3] and high MSS prevalence in specific occupational groups such as teachers [7,23]. However, these studies have not specifically examined, at the population level, whether measured BMI is associated with both the overall number of recent non-traumatic MSS and their anatomical distribution across multiple body regions. In addition, evidence addressing this association in nationally representative Latin American populations remains limited. Therefore, the present study aimed to analyze the association between BMI and the prevalence and burden of MSS in the Chilean population using data from the 2009–2010 National Health Survey and accounting for its complex sampling design. We hypothesized that higher BMI would be positively associated with a greater prevalence and burden of MSS.

## 2. Materials and Methods

### 2.1. Study Population

The data collected by the NHS between October 2009 and September 2010 were used [25]. The 2002 Population and Housing Census defined the representative sample as national, regional, and rural/urban. The sample design was a stratified multistage design calculated with a relative error of 20%. It was focused on people aged 15 years or older. Of the 5293 participants in the 2009–2010 Chilean NHS database, 4689 were included in the final analytic sample. The participant selection process is summarized in Figure 1. Details regarding missing data and the complete-case analytical approach are provided below.

#### Missing Data

Missing data were present for several variables required for the analysis. Of the 5293 participants in the original survey dataset, 489 had missing BMI data, 97 did not have a valid survey expansion factor, 16 had missing educational-level data, and 2 lacked information required for the regional MSS analysis. These participants were excluded sequentially, resulting in a final analytic sample of 4689 participants. A complete-case analytical approach was used for BMI, educational level, musculoskeletal outcomes, and survey-design variables. No missing-data imputation was performed.

The sociodemographic characteristics considered in this study are age, sex (male or female; as recorded in the NHS), educational level, and area of residence (urban or rural). Age was grouped into four categories, continuing with the categorization used in the 2009–2010 NHS (15–24 years, 25–44 years, 45–64 years, and 65 years and over). The educational level variable has three groups: “low” (less than 8 years of study), “medium” (8–12 years of study), and “high” (more than 12 years of study).

### 2.2. Instruments

#### 2.2.1. Body Mass Index

The 2009–2010 NHS used anthropometry to assess nutritional status, including weight and height measurements. BMI was calculated as weight (kg) divided by height squared (m^2^). For this study, the parameters established by the National Heart, Lung and Blood Institute and the North American Association for the Study of Obesity were considered for the BMI categories: persons with BMI < 25 kg/m^2^, persons with BMI 25 to <30 kg/m^2^ (overweight), and persons with BMI ≥ 30 kg/m^2^ (obesity) [26]. The BMI < 25 kg/m^2^ reference category included both participants with underweight (BMI < 18.5 kg/m^2^) and those within the normal-weight range (BMI 18.5 to <25 kg/m^2^). These BMI thresholds were applied consistently across the analytic sample. This categorization was used to contrast participants without excess body weight with those classified as overweight or obese.

#### 2.2.2. Musculoskeletal Symptoms

The 2009–2010 NHS used the results of the COPCORD CCQ-ILAR (Community Oriented Programme for the Control of Rheumatic Diseases) questionnaire, which was translated, adapted, and validated in Spanish in the survey, to screen for MSS of non-traumatic origin in the “last seven days”. The questionnaire was used to identify self-reported MSS and was not intended to establish clinical diagnoses of specific musculoskeletal disorders. This questionnaire had a specificity of 76% and a sensitivity of 92%. In the present study, MSS of non-traumatic origin reported during the previous seven days were analysed according to their presence and anatomical distribution. Pain intensity was not included in the available outcome definition; therefore, the analyses reflect symptom occurrence and the number of affected anatomical regions rather than symptom severity. The body segments considered for the analysis are the neck, shoulder, upper back, lower back, elbow, wrist, fingers of the hand, hip, knee, ankle, toes, or another joint. Total MSS was defined as the sum of the anatomical regions in which non-traumatic MSS were reported. Because 12 anatomical regions were assessed, the total MSS count ranged from 0 to 12, with higher values indicating a greater number of symptomatic regions.

### 2.3. Statistical Analysis

Statistical analyses used Stata Statistical Software, version 16, for Windows (StataCorp LLC, College Station, TX, USA). All analyses incorporated the complex sampling design of the 2009–2010 Chilean National Health Survey by applying sampling weights, strata, and primary sampling units. The survey weight corresponding to the anthropometric examination component was used because the analyses combined measured BMI with musculoskeletal outcomes. Sample characteristics were described using unweighted frequencies, weighted percentages, and medians with interquartile ranges, as appropriate.

The association between BMI and the total number of musculoskeletal symptoms (total MSS) was evaluated using a survey-weighted negative binomial regression model. This approach was selected because total MSS was a non-negative count variable and exhibited overdispersion, making the negative binomial model more appropriate than a Poisson model. The total MSS count was retained as the primary outcome. For complementary analyses, two dichotomous outcomes were additionally examined: reporting at least one MSS (≥1 MSS) and at least two MSS (≥2 MSS), corresponding to the median and 75th percentile of the observed MSS-count distribution, respectively. These thresholds were not intended to represent clinically validated cut-off points [7,27]. These logistic regression models [28] complemented the negative binomial model by estimating the odds of belonging to each symptom-burden category.

Region-specific associations were evaluated by fitting separate survey-weighted logistic regression models for each anatomical region. In each model, the presence of musculoskeletal symptoms in a single anatomical region served as the dependent variable, and BMI category was the primary independent variable. Models for the neck, shoulder, upper back, lower back, elbow, wrist, fingers, hip, knee, ankle, toes, and other joints were estimated independently. All regression models were adjusted for age, educational level, sex, and area of residence.

Results from the negative binomial regression model are presented as count ratios (CRs), obtained by exponentiating the regression coefficients and interpreted as the ratio of expected MSS counts. Results from the logistic regression models are reported as odds ratios (ORs) with 95% confidence intervals (CIs). For the region-specific analyses, the 24 *p*-values corresponding to two BMI contrasts across 12 anatomical regions were adjusted using the Benjamini–Hochberg false discovery rate (FDR) procedure. Statistical significance for these comparisons was defined as an FDR-adjusted *p*-value < 0.05. Goodness of fit for each survey-weighted logistic regression model was assessed using the F-adjusted Archer–Lemeshow test. The goodness-of-fit *p*-values were not included in the multiple-comparison correction.

## 3. Results

The total sample consisted of 4689 participants with complete data. Table 1 shows the distribution of BMI categories, with 39.1% classified as overweight (25 to <30 kg/m^2^) and 25.3% as obese (≥30 kg/m^2^). Among the 1447 participants in the BMI < 25 kg/m^2^ reference category, 79 had BMI < 18.5 kg/m^2^, corresponding to 5.46% of this group in the unweighted sample and 4.86% after survey weighting (95% CI: 3.40–6.90%). It also shows that the BMI category was significantly associated with sex, age group, educational level, and the presence of MSS (*p* < 0.001). Regarding sex and BMI categories, men represented a greater proportion of the overweight category than women (55.6% vs. 44.4%), whereas women represented a greater proportion of the obesity category (62.8% vs. 37.2%). The global median number of MSS was 1 (IQR: 0–2), with higher counts among participants with overweight or obesity.

Figure 2 shows the weighted prevalence of MSS by body region and BMI category. Across all three BMI groups, the knee, lower back, shoulder, and ankle were the most affected regions. Among the anatomical segments studied, it is interesting to note the high prevalence of MSS in the knee and the increase in MSS with increasing BMI: from 17.1% in the BMI < 25 kg/m^2^ group to 25.4% in the overweight group and 34.9% in the obese group. Shoulder symptoms showed a similar trend, increasing from 9.1% to 14.5% and 21.7%, while ankle symptoms rose from 6.5% to 10.3% and 16.8%. Regarding lower back symptoms, MSS increased from 13.7% in participants with BMI < 25 kg/m^2^ to 20.4% in those who are overweight and 20.7% in participants classified as obese. Finger symptoms also showed an increase across BMI categories: 7.1% for BMI < 25 kg/m^2^, 12.2% for overweight, and 18.1% for obesity.

Regarding the association between BMI and the total MSS count (Table 2), each one-unit increment in BMI corresponded to a 2.8% increase in the expected symptom count (CR = 1.028; 95% CI: 1.019–1.037; *p* < 0.001), after adjustment for age, educational level, sex, and area of residence. Although the effect per BMI unit was modest, the same association expressed over a 5 kg/m^2^ increment corresponded to an approximately 14.8% higher expected MSS count (CR ≈ 1.148; 95% CI ≈ 1.099–1.199). Age (CR = 1.016; 95% CI: 1.012–1.020; *p* < 0.001) and female sex (vs. male; CR = 1.508; 95% CI: 1.334–1.705; *p* < 0.001) were also independently associated with greater MSS burden. In the adjusted model, educational level and area of residence were not statistically significant.

A complementary set of logistic regression models was fitted using two dichotomized versions of the total MSS count, based on the 50th and 75th percentiles (Table 3). An analysis of BMI categories reveals that participants with overweight or obesity had higher odds of reporting MSS. In this sense, for the *p*50 cut-off (total MSS ≥ 1), participants with overweight had 55.7% higher odds of reporting at least one MSS than those with BMI < 25 kg/m^2^ (OR = 1.557; 95% CI: 1.215–1.996; *p* < 0.001), whereas participants with obesity had 98.4% higher odds (OR = 1.984; 95% CI: 1.524–2.583; *p* < 0.001). Age and sex were also significant factors in the models, whereas educational level and area of residence were not.

Region-specific logistic regression models showed heterogeneous associations between BMI categories and MSS across anatomical regions. After correction for multiple comparisons, overweight remained significantly associated with lower-back and finger symptoms, whereas the association with shoulder symptoms did not remain statistically significant. Full effect estimates, confidence intervals, and adjusted *p*-values are presented in Table 4.

Obesity remained significantly associated with shoulder, wrist, and finger symptoms after correction for multiple comparisons, whereas the associations with neck, lower-back, and elbow symptoms did not remain statistically significant. Upper-back symptoms were not significantly associated with either BMI category. Detailed estimates for these associations are provided in Table 4.

Among the lower-body regions, overweight remained significantly associated with knee symptoms after correction for multiple comparisons, whereas the association with ankle symptoms did not remain statistically significant. Obesity remained significantly associated with knee and ankle symptoms, whereas the association with hip symptoms did not remain statistically significant after correction. No statistically significant associations were observed for toe symptoms or other joint symptoms. The toe-symptom model showed evidence of inadequate fit and should therefore be interpreted cautiously. Detailed results are presented in Table 5.

## 4. Discussion

This study identified a positive association between BMI and MSS burden in the Chilean population. MSS prevalence increased across BMI categories and was highest among participants with obesity, while the knee showed the steepest regional gradient. Approximately 64.4% of respondents had a BMI ≥25 kg/m^2^ and about 25% had a BMI ≥30 kg/m^2^. These findings should be interpreted in the context of the 2009–2010 survey period; in particular, the prevalence estimates describe the population at that time and should not be interpreted as representing the current prevalence of MSS or obesity in Chile. Women represented a larger proportion of the obesity category, consistent with previous international evidence [29,30]. Likewise, the population aged 45–64 years presented high levels of overweight and obesity compared to the other age categories. In this regard, other studies indicate a significant association between increasing age and obesity [31,32,33]. In particular, in the Chilean population, a sustained increase in the prevalence of obesity has been observed through national health surveys [24], which indicates that obesity remains a serious public health problem and affects other systems, such as the musculoskeletal system.

The main results reported that more than half of the Chilean population surveyed (55.26%) presented MSS. When comparing the prevalence of MSS in other populations, such as that reported by a study of the South Korean population [34] with 38.3% of a total of 1576 participants manifesting MSS associated with daily life, or in Brazilians, where 26.9% of respondents reported MSS not associated with trauma [35], we have that in Chile the prevalence of MSS for the general population is high. However, other studies focused on the working population report an even higher prevalence of MSS, such as in New Zealand, where 92% of respondents (*n* = 3003) reported MSS in any body region [36], or in the Spanish working population, where almost 89% of participants reported MSS [37]. Direct comparisons should be interpreted cautiously because the populations, recall periods, symptom definitions, and instruments differed across studies.

Although the present investigation found a higher prevalence of MSS in the lower limb, a review of the related international literature revealed differences in MSS prevalence by body zone across worker groups. For example, in Chilean teachers, high prevalence of MSS (91%) was reported, higher than those found in the present investigation, mainly in segments such as the lower back, neck, and shoulders [7]. On the other hand, Australian and Danish workers who use computers for long working hours manifest more MSS in the neck, shoulder, elbow, or wrist [38,39], similar to what was found in dentists or dental assistants, who report mostly neck discomfort [40]. In workers in the industrial sector (e.g., packing), MSS occur more frequently in the lower back, shoulders, and neck [41]. Another study focused on Thai office workers reported a higher prevalence of MSS in the neck, shoulders, upper back, and ankles in women than in men [42], and office workers younger than 30 years were more likely to present upper back symptoms compared to those older than 49 years. Italian health professionals [43] report a high prevalence of low back MSS. However, in female nurses, there is a double risk of presenting with upper back and neck pain compared to men. These investigations show an essential relationship between MSS and the work performed, suggesting that the high prevalence of MSS reported in the Chilean population could increase with occupational factors, making it relevant to investigate professional groups at risk of MSS.

Age and female sex were independently associated with a greater overall MSS burden, consistent with previous population-based evidence showing a higher prevalence of MSS among women [36,44].

Regarding BMI and MSS, it was observed that participants with obesity showed the highest prevalence of MSS (67%), compared to the overweight group (BMI 25 to <30 kg/m^2^; 58%) and the group with BMI less than 25 kg/m^2^ (44.7%); this indicates that the prevalence of MSS increases as BMI increases. These prevalences are higher than those reported previously [5], where 50% of those with normal weight according to BMI presented MSS, 52.3% of overweight participants, and 57.6% of respondents with obesity. In addition, the present study found that higher BMI was associated with a greater overall MSS burden and with selected region-specific symptoms, particularly in weight-bearing lower-limb regions. Although the association between continuous BMI and total MSS burden was statistically robust, its magnitude was modest on a per-unit basis: each 1 kg/m^2^ increase in BMI was associated with a 2.8% higher expected MSS count. When expressed over a 5 kg/m^2^ increment, the same association corresponded to an approximately 14.8% higher expected symptom count, providing a more interpretable indication of the cumulative magnitude of the association across larger differences in BMI.

The knee region exhibited the highest prevalence of MSS across all BMI categories. Specifically, the prevalence was 17.1% in participants with a BMI below 25 kg/m^2^, 25.4% in those with overweight, and 34.9% in individuals with obesity. This represented the steepest increase among the anatomical regions analyzed. After correction for multiple comparisons, overweight remained significantly associated with symptoms in the lower back, fingers, and knee, whereas obesity remained associated with symptoms in the shoulder, wrist, fingers, knee, and ankle. The knee showed the most consistent pattern, remaining significantly associated with both overweight and obesity, while the ankle association was retained only for obesity. These findings are broadly consistent with previous studies [2,5,45,46,47,48,49,50] reporting greater lower-limb musculoskeletal burden, particularly knee symptoms, among individuals with elevated BMI. Several nominal associations, including those involving the neck, elbow, hip, and selected shoulder, lower-back, and ankle contrasts, did not remain statistically significant after FDR correction and should therefore be interpreted cautiously.

The observed regional pattern is biologically compatible with both mechanical and systemic pathways, although these mechanisms were not directly assessed in the present study. Excess body weight may increase mechanical loading in weight-bearing regions such as the knee and ankle, potentially contributing to greater musculoskeletal stress [13,14,15,16,17,18,51,52,53]. This interpretation is consistent with the stronger associations observed in lower-limb regions, particularly the knee. However, the cross-sectional design does not allow determination of whether increased BMI preceded MSS or whether pain-related reductions in mobility may have contributed to higher BMI.

The associations observed in non-weight-bearing regions, including the shoulder, wrist, and fingers, suggest that mechanisms beyond mechanical loading may also be relevant. Obesity-related systemic inflammatory and metabolic processes have been proposed as potential contributors to musculoskeletal symptoms [54,55,56,57,58,59]. Nevertheless, these pathways were not measured in this study and should therefore be considered biologically plausible contextual explanations rather than mechanisms demonstrated by the present findings. Residual confounding by physical activity, occupational workload, and comorbid conditions also cannot be excluded.

BMI should nevertheless be interpreted as an anthropometric proxy rather than a direct measure of adiposity or body composition. Individuals with similar BMI values may differ considerably in total and regional fat mass, visceral adiposity, and lean body mass [21,22]. Moreover, individuals within the conventional normal-BMI range may present excess body fat, a phenotype commonly described as normal-weight obesity. Recent evidence has shown that such individuals may have greater visceral adiposity and lower lean mass despite having BMI values within the normal range [60]. Similarly, associations between body-composition parameters and musculoskeletal characteristics may differ according to sex and obesity status [61]. Therefore, the mechanical and metabolic pathways discussed below should not be attributed to BMI itself, but rather considered as biologically plausible pathways related to the body-composition characteristics that BMI only indirectly reflects.

### Limitations

Although BMI is widely adopted by the National Institutes of Health and the World Health Organization, it does not account for body composition, body fat distribution, or age and sex [62], which is a limitation for this study. In addition, the BMI < 25 kg/m^2^ reference category included both normal-weight and underweight participants. Although participants with BMI < 18.5 kg/m^2^ represented only 4.86% of this reference group after survey weighting, combining these categories may introduce some heterogeneity into the reference comparison. Another limitation of the study is that the pain intensity associated with BMI was not evaluated, which may have affected the results for joints with a higher prevalence of MSS. A further limitation concerns the model fit for toe symptoms, which the test indicated was inadequate; consequently, the associations reported for this region are considered exploratory and should be interpreted cautiously. Another limitation is the age of the data source. This study used data from the 2009–2010 Chilean National Health Survey, and the distribution of BMI and MSS in the Chilean population may have changed since then. Therefore, the prevalence estimates reported here should not be interpreted as reflecting the current burden of MSS in Chile. Nevertheless, the use of a nationally representative survey with standardized anthropometric and musculoskeletal assessments provides valuable population-based evidence on the association between BMI and MSS burden. Future studies using more recent national data are needed to evaluate whether these associations persist under the current epidemiological profile of obesity in Chile. In addition, MSS were self-reported, which could introduce bias, as participants may not accurately recall it. Residual confounding cannot be excluded because the 2009–2010 Chilean NHS did not include detailed measures of occupational or physical workload, and objective physical activity assessment by accelerometry was not part of this survey. Because a complete-case analytical approach was used, selection bias due to missing data cannot be entirely excluded, particularly if missingness was associated with BMI, MSS, or other participant characteristics. No missing-data imputation was performed. Future studies should also investigate whether additional demographic and occupational factors, such as type of employment, are associated with MSS in specific anatomical regions, particularly to identify occupational groups at increased risk in the Chilean population.

## 5. Conclusions

In summary, higher BMI was positively associated with a greater overall MSS burden in the Chilean population included in the 2009–2010 National Health Survey. Region-specific analyses identified the most robust associations in the lower back, fingers, and knee among participants with overweight, and in the shoulder, wrist, fingers, knee, and ankle among participants with obesity, after correction for multiple comparisons. Other nominal associations that did not remain statistically significant after correction should be interpreted cautiously. These findings demonstrate associations rather than causal relationships. Given the cross-sectional design, causal directionality cannot be established, and reverse or bidirectional relationships should be considered. Future longitudinal studies using more recent nationally representative data are needed to confirm these findings and evaluate their relevance under the current epidemiological profile of obesity in Chile.

## Figures and Tables

**Figure 1:**
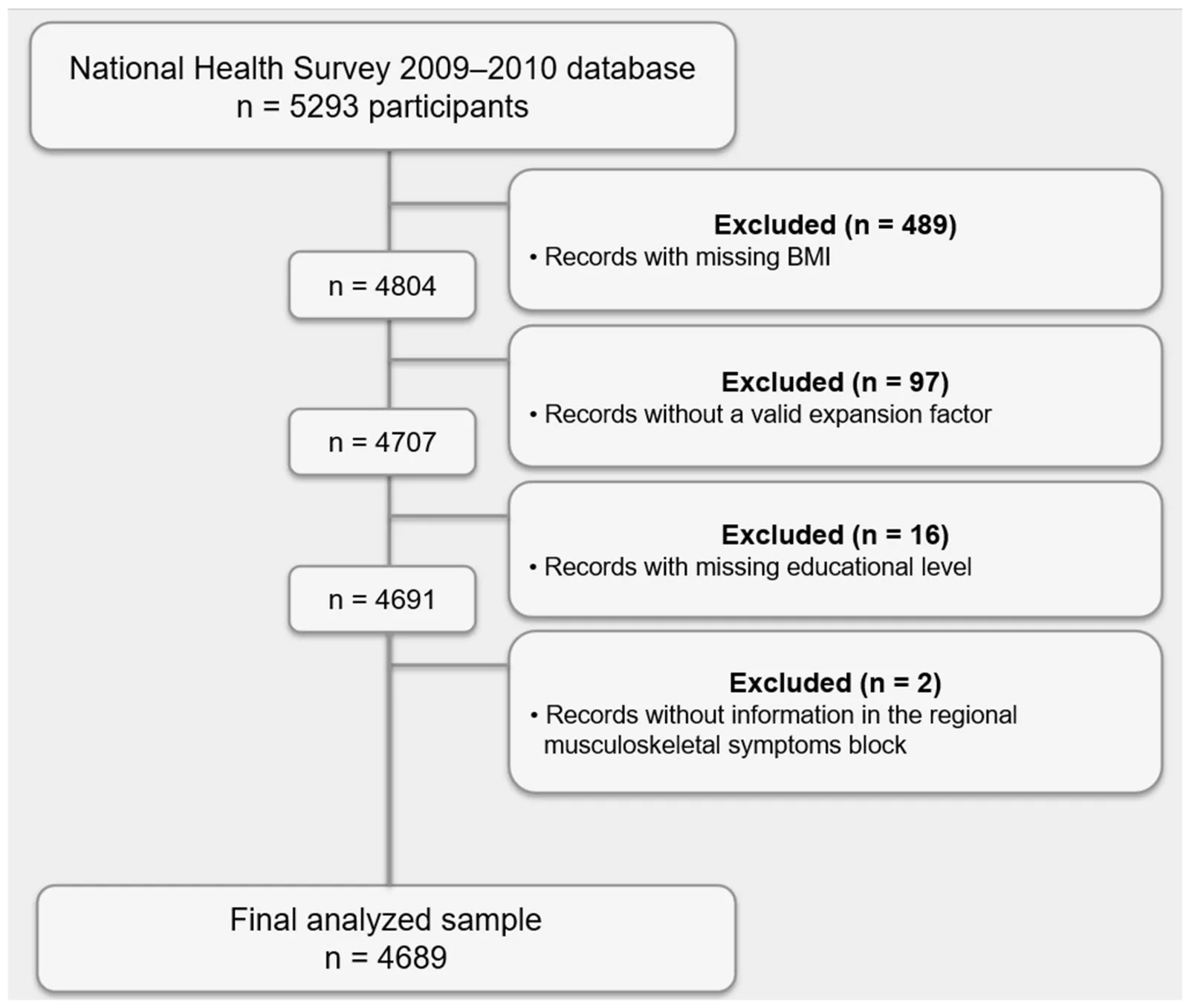
Flowchart of participant selection from the 2009–2010 Chilean National Health Survey (n = 5293) to the final analytic sample (n = 4689).

**Figure 2:**
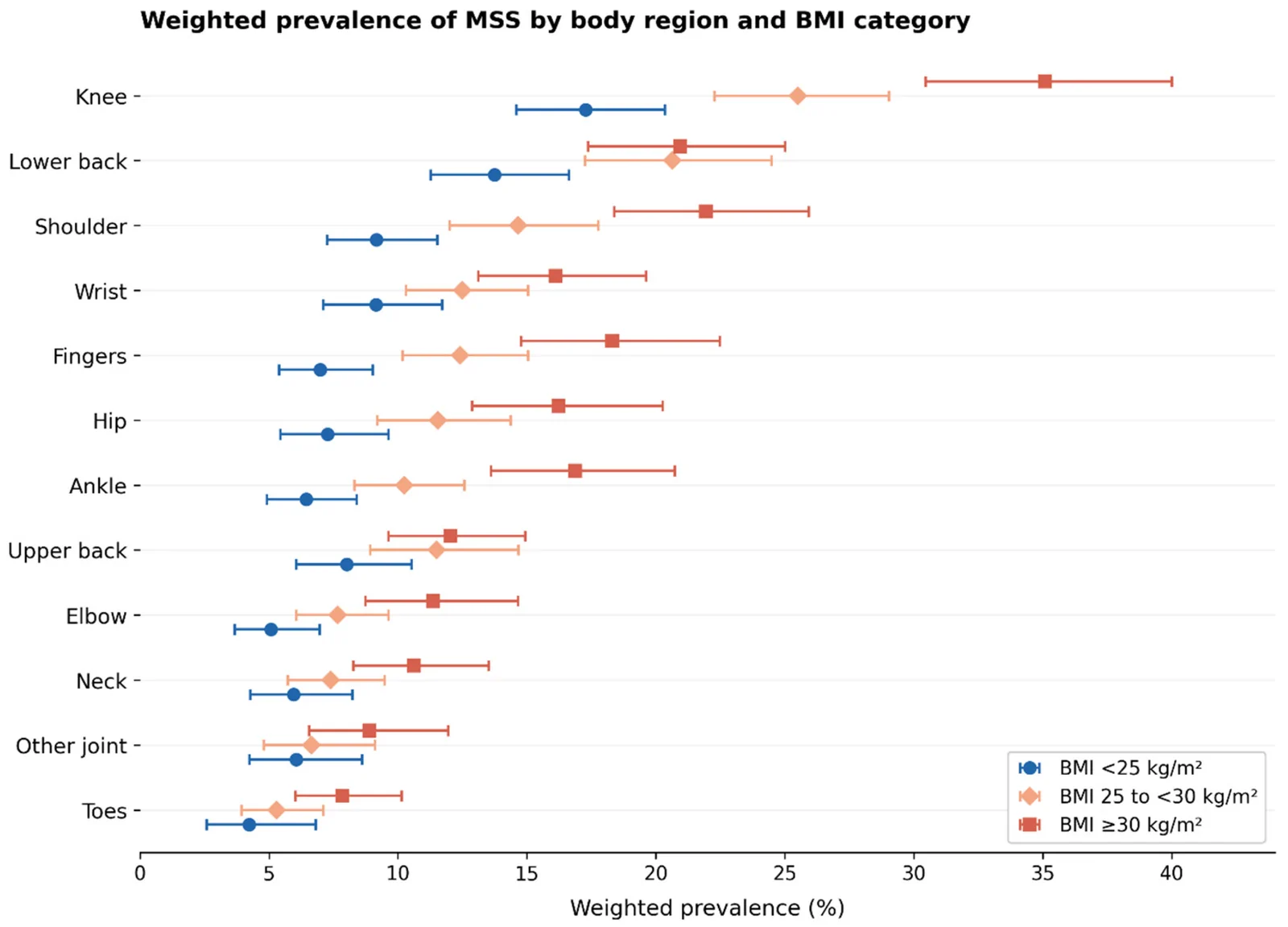
Weighted prevalence of musculoskeletal symptoms by body region according to BMI categories in the analytic sample of the 2009–2010 Chilean National Health Survey (n = 4689). Points represent weighted prevalence estimates and whiskers indicate 95% confidence intervals.

**Table 1:** Sociodemographic characteristics and musculoskeletal symptoms according to body mass index categories in the analytic sample of the 2009–2010 Chilean National Health Survey (n = 4689).

Variable	Total n (Weighted %)	BMI < 25 n (Weighted %)	BMI 25 to <30 n (Weighted %)	BMI ≥ 30 n (Weighted %)	*p*-Value ^1^
Total	4689 (100.0)	1447 (35.65)	1874 (39.10)	1368 (25.26)	—
Sex					<0.001
Male	1885 (48.31)	585 (48.21)	857 (55.57)	443 (37.22)	
Female	2804 (51.69)	862 (51.79)	1017 (44.43)	925 (62.78)	
Age group (years)					<0.001
15–24	702 (22.08)	423 (38.56)	193 (15.23)	86 (9.40)	
25–44	1569 (37.57)	480 (34.59)	650 (41.36)	439 (35.93)	
45–64	1553 (28.92)	326 (18.45)	678 (30.93)	549 (40.59)	
≥65	865 (11.43)	218 (8.40)	353 (12.49)	294 (14.08)	
Educational level					<0.001
Low	1220 (18.10)	291 (12.10)	483 (19.10)	446 (25.03)	
Medium	2579 (57.49)	833 (61.72)	1014 (54.22)	732 (56.57)	
High	890 (24.41)	323 (26.18)	377 (26.69)	190 (18.40)	
Area of residence					0.268
Urban	4001 (87.15)	1268 (88.64)	1592 (86.97)	1141 (85.30)	
Rural	688 (12.85)	179 (11.36)	282 (13.03)	227 (14.70)	
Any MSS in the last 7 days					<0.001
No	2018 (44.74)	743 (55.34)	793 (42.41)	482 (33.38)	
Yes	2671 (55.26)	704 (44.66)	1081 (57.59)	886 (66.62)	
Total number of MSS, median (IQR)	1 (0–2)	0 (0–2)	1 (0–2)	1 (0–2)	—

Values are presented as unweighted n and weighted percentages, unless otherwise indicated. BMI, body mass index; IQR, interquartile range; MSS, musculoskeletal symptoms, defined as the presence of any non-traumatic musculoskeletal symptom during the 7 days before the survey. The analytic sample included participants with complete information on musculoskeletal outcomes, body mass index, covariates, and complex survey design variables. ^1^ *p*-Values were obtained using design-based tests accounting for the complex sampling design of the survey. For categorical variables, survey-adjusted Pearson tests were used. For the total number of MSS, the median (IQR) is presented descriptively; no *p*-value is shown.

**Table 2:** Negative binomial regression model for the number of musculoskeletal symptoms according to body mass index and covariates, accounting for the complex sampling design.

Variable	Adjusted CR	95% CI	*p*-Value
BMI (kg/m^2^)	1.028	1.019–1.037	<0.001
Age (years)	1.016	1.012–1.020	<0.001
Medium educational level vs. low	0.940	0.807–1.095	0.430
High educational level vs. low	0.835	0.671–1.038	0.105
Female vs. male	1.508	1.334–1.705	<0.001
Rural vs. urban	1.061	0.898–1.253	0.490

Results are presented as incidence count ratios (CR) with 95% confidence intervals (CIs). Reference categories were low educational level, male sex, and urban residence. BMI, body mass index.

**Table 3:** Logistic regression models for total musculoskeletal symptoms using *p*50 and *p*75 cut-off points, with categorical body mass index and accounting for the complex sampling design.

Variable	Adjusted OR (*p*50)	95% CI	*p*-Value	Adjusted OR (*p*75)	95% CI	*p*-Value
BMI 25 to <30 vs. <25 kg/m^2^	1.557	1.215–1.996	<0.001	1.534	1.178–1.998	0.002
BMI ≥ 30 vs. <25 kg/m^2^	1.984	1.524–2.583	<0.001	1.931	1.470–2.537	<0.001
Age (years)	1.014	1.008–1.021	<0.001	1.019	1.013–1.026	<0.001
Medium educational level vs. low	0.879	0.673–1.149	0.346	0.966	0.711–1.313	0.826
High educational level vs. low	0.730	0.515–1.035	0.077	0.782	0.535–1.144	0.206
Female vs. male	1.577	1.281–1.940	<0.001	1.713	1.365–2.149	<0.001
Rural vs. urban	1.036	0.771–1.391	0.814	0.878	0.662–1.164	0.367

Survey-weighted logistic regression models were fitted using two dichotomous outcomes derived from the total number of musculoskeletal symptoms (MSS): *p*50 (total MSS ≥ 1) and *p*75 (total MSS ≥ 2). All models were adjusted for body mass index (BMI) categories, age, educational level, sex, and area of residence, accounting for the complex sampling design of the survey. Results are presented as adjusted odds ratios (ORs) with 95% confidence intervals (CIs). Reference categories were BMI < 25 kg/m^2^, low educational level, male sex, and urban residence.

**Table 4:** Adjusted associations between body mass index categories and musculoskeletal symptoms in the neck, upper limb, and back regions.

Body Region	BMI 25 to <30 vs. <25 kg/m^2^ OR (95% CI)	FDR-Adjusted *p*-Value	BMI ≥ 30 vs. <25 kg/m^2^ OR (95% CI)	FDR-Adjusted *p*-Value	Archer–Lemeshow *p*-Value
Neck	1.253 (0.784–2.000)	0.377	1.619 (1.019–2.570)	0.071	0.756
Shoulder	1.479 (1.042–2.098)	0.057	2.072 (1.454–2.953)	<0.001	0.663
Upper back	1.464 (0.962–2.227)	0.112	1.322 (0.886–1.971)	0.228	0.761
Lower back	1.599 (1.164–2.196)	0.016	1.493 (1.063–2.096)	0.052	0.958
Elbow	1.321 (0.855–2.039)	0.265	1.685 (1.079–2.630)	0.052	0.802
Wrist	1.398 (0.971–2.012)	0.112	1.590 (1.096–2.307)	0.044	0.809
Fingers	1.721 (1.188–2.492)	0.016	2.195 (1.471–3.276)	<0.001	0.195

Survey-weighted logistic regression models were fitted separately for each anatomical region. All models were adjusted for age, educational level, sex, and area of residence. Results are presented as adjusted odds ratios (ORs) with 95% confidence intervals (CIs). Reference categories were BMI < 25 kg/m^2^, low educational level, male sex, and urban residence. The *p*-values for the 24 BMI-related comparisons, corresponding to two BMI contrasts across 12 anatomical regions, were adjusted using the Benjamini–Hochberg false discovery rate (FDR) procedure. Statistical significance was defined as an FDR-adjusted *p*-value < 0.05. The Archer–Lemeshow *p*-values were used exclusively to assess model goodness of fit and were not included in the multiple-comparison correction. Model fit was adequate for the neck, shoulder, upper-back, lower-back, elbow, wrist, and fingers models according to the F-adjusted Archer–Lemeshow test. BMI, body mass index. Confidence intervals are presented for reference and were not adjusted for multiple comparisons.

**Table 5:** Adjusted associations between body mass index categories and musculoskeletal symptoms in the lower body regions and other joints.

Body Region	BMI 25 to <30 vs. <25 kg/m^2^ OR (95% CI)	FDR-Adjusted *p*-Value	BMI ≥ 30 vs. <25 kg/m^2^ OR (95% CI)	FDR-Adjusted *p*-Value	Archer–Lemeshow *p*-Value
Hip	1.440 (0.947–2.189)	0.124	1.648 (1.063–2.555)	0.056	0.219
Knee	1.418 (1.077–1.867)	0.044	1.940 (1.424–2.642)	<0.001	0.907
Ankle	1.492 (1.031–2.160)	0.063	2.268 (1.519–3.386)	<0.001	0.623
Toes	1.022 (0.551–1.896)	0.944	1.374 (0.780–2.418)	0.325	<0.001
Other joint	1.028 (0.603–1.753)	0.944	1.298 (0.801–2.102)	0.330	0.225

Survey-weighted logistic regression models were fitted separately for each anatomical region. All models were adjusted for age, educational level, sex, and area of residence. Results are presented as adjusted odds ratios (ORs) with 95% confidence intervals (CIs). Reference categories were BMI < 25 kg/m^2^, low educational level, male sex, and urban residence. The *p*-values for the 24 BMI-related comparisons, corresponding to two BMI contrasts across 12 anatomical regions, were adjusted using the Benjamini–Hochberg false discovery rate (FDR) procedure. Statistical significance was defined as an FDR-adjusted *p*-value < 0.05. The Archer–Lemeshow *p*-values were used exclusively to assess model goodness of fit and were not included in the multiple-comparison correction. Model fit was adequate for the hip, knee, ankle, and other joint models, whereas the toes model showed evidence of lack of fit according to the F-adjusted Archer–Lemeshow test. BMI, body mass index. Confidence intervals are presented for reference and were not adjusted for multiple comparisons.

## Data Availability

The data underlying the results presented in this study are publicly available from the Chilean Ministry of Health at https://epi.minsal.cl/resultados-encuestas/ (accessed on 5 August 2026).

## Abbreviations

The following abbreviations are used in this manuscript:

BMI	Body mass index
CI	Confidence interval
COPCORD	Community Oriented Programme for the Control of Rheumatic Diseases
FDR	False discovery rate
IQR	Interquartile range
CR	Count ratio
MSS	Musculoskeletal symptoms
NHS	National Health Survey
OR	Odds ratio
